# Classification of deep vein thrombosis stages using convolutional neural network of electromyogram with vibrotactile stimulation toward developing an early diagnostic tool: A preliminary study on a pig model

**DOI:** 10.1371/journal.pone.0281219

**Published:** 2023-02-02

**Authors:** Jong Woo Kang, Keun-Tae Kim, Jong Woong Park, Song Joo Lee

**Affiliations:** 1 Department of Orthopaedic Surgery, Korea University Ansan Hospital, Ansan, Republic of Korea; 2 Bionics Research Center, Korea Institute of Science and Technology, Seoul, Republic of Korea; 3 Department of Orthopaedic Surgery, Korea University Anam Hospital, Seoul, Republic of Korea; 4 Division of Bio-Medical Science & Technology, KIST School, Korea University of Science and Technology, Seoul, Republic of Korea; Justus Liebig University Giessen, GERMANY

## Abstract

Deep vein thrombosis (DVT) can lead to life-threatening disorders; however, it can only be recognized after its symptom appear. This study proposed a novel method that can detect the early stage of DVT using electromyography (EMG) signals with vibration stimuli using the convolutional neural networks (CNN) algorithm. The feasibility of the method was tested with eight legs before and after the surgical induction of DVT at nine-time points. Furthermore, perfusion pressure (PP), intracompartmental pressure (IP), and shear elastic modulus (SEM) of the tibialis anterior were also collected. In the proposed method, principal component analysis (PCA) and CNN were used to analyze the EMG data and classify it before and after the DVT stages. The cross-validation was performed in two strategies. One is for each leg and the other is the leave-one-leg-out (LOLO), test without any predicted information, for considering the practical diagnostic tool. The results showed that PCA-CNN can classify before and after DVT stages with an average accuracy of 100% (each leg) and 68.4±20.5% (LOLO). Moreover, all-time points (before induction of DVT and eight-time points after DVT) were classified with an average accuracy of 72.0±11.9% which is substantially higher accuracy than the chance levels (11% for 9-class classification). Based on the experimental results in the pig model, the proposed CNN-based method can classify the before- and after-DVT stages with high accuracy. The experimental results can provide a basis for further developing an early diagnostic tool for DVT using only EMG signals with vibration stimuli.

## Introduction

Deep vein thrombosis (DVT) occurs during certain medical conditions such as hypercoagulability, venous stasis, and changes in endothelial lining [1–3]. Also, it can occur spontaneously even during a long-time drive or long-distance flight. The annual incidence of DVT is 67 per 100,000 among the general population in the USA [4]. DVT can cause life-threatening disorders such as chronic thromboembolic pulmonary hypertension, pulmonary thromboembolism, and post-thrombotic syndrome [5]. In addition, it has a considerable economic impact on patients [6].

The current protocol for diagnosing DVT is to use a duplex ultrasound or venography even after recognizing clinical signs and symptoms of DVT (such as swelling and tightness) in an affected limb [1,7]. Otherwise, a D-dimer test is performed for DVT high-risk patients [1,2,7]. Nevertheless, early recognition of DVT is important in reducing its mortality [2,8]. However, the need of performing diagnostic examinations can be easily neglected in silent DVT limbs. Furthermore, it is also impossible to perform to diagnose DVT outside of the hospital during a long-time drive or long-distance flight. D-dimer levels are elevated not only in the setting of acute thrombosis, but also in other conditions such as pregnancy, infection, cancer, or surgery [1,2]. Especially, surgery is a representative cause of a false positive D-dimer test in the postoperative period [9]. That is not helpful and can lead to confusion in diagnosing DVT. Consequently, prophylaxis with low-dose anticoagulation or pneumatic compression or compression stockings is performed for patients with risk factors such as prolonged bed rest, estrogen, congestive heart failure, family history, hematologic cancers, major trauma, obesity, pregnancy (and postpartum status), solid cancers, recent surgery, and stroke [1,7]. DVT prophylaxis can cause adverse effects such as major hemorrhage and cannot be administered to some patients [10]. Furthermore, the effect of prophylaxis is sometimes incomplete [10]. The ideal way to prevent the danger of DVT is to continuously monitor early physiologic changes in DVT before its development and understanding the physiologic changes is essential for DVT monitoring [11]. However, there is a lack of monitoring tools for the early physiologic changes of DVT everywhere, and the early physiologic changes remain unclear.

Venous stasis in the affected limb due to DVT could lead to a change in muscle stiffness. Although duplex ultrasonography has been a popular method of detecting DVT [12], it may be almost impossible to attach the ultrasound probe for a long enough time to detect the early stage of DVT, and also impossible to use outside clinics. As an alternative method of measuring muscle stiffness, the mechanical response of the muscle can be evoked by a mechanical impulse or vibration using an accelerometer [13] or electromyography (EMG) [14]. When a vibration stimulation is applied to the skin of the target muscle, a change in the EMG can also occur due to associated changes in the stiffness of the skin and muscles in a relaxed state [15]. Furthermore, physiological events associated with venous congestion in extremities related to DVT can change EMG signals. Colombo et al. reported in their experimental studies for physiologic change during acute venous congestion, venous neurohormones levels in extremities are significantly increased at 75 min of venous congestion and Shimabukuro et al. reported that venous sodium ion in extremities is significantly decreased at 30 min of venous congestion [16,17]. Barrett-O’Keefe et al. said that endothelin-1 (ET-1) has an important role in the regulation of skeletal muscle blood flow as a vasoconstrictor [18]. Lin et al. reported that the blood level of ET-1 is continuously increased in venous congestion at least 2 hours after induction of venous congestion [19]. Also, it is well-known that extremity venous congestion elevates blood calcium levels [20]. Therefore, to diagnose or detect the early stage of DVT, changes in EMG with vibration stimuli can be monitored.

The use of pattern recognition and machine learning (PRML) techniques is increasing in recent studies on the prediction, diagnosis, classification, and early detection of disease in medicine and biomedical engineering [21–24]. Specifically, random forest [21], decision trees [25], the support vector machine [26], or convolutional neural networks (CNN) [27] have been applied to the classification, diagnosis, and early detection of disease with excellent results [22]. In particular, some studies have reported the efficacy and reliability of PRML techniques for predicting and diagnosing thromboembolic disease [28–33]. However, PRML techniques have not yet been applied to the early diagnosis of DVT. If the changes can be detected via CNN-based EMG analysis, we hypothesize that early diagnosis or detection of the initial stage of DVT would be possible. CNN has recently become one of the major approaches among the PRML techniques. Following the advances in computing power achieved through the development of graphics processors (GPUs), CNN has been applied to image and signal processing in previous studies [34,35]. However, to our best knowledge, CNN has not been applied to early diagnosis methods for DVT or detecting the initial stage of DVT. Especially, in this study, we focus on the early diagnosis of more clinically significant and serious forms of DVT (completely obstructive symptomatic proximal DVT).

Thus, the goal of our study was to develop a new method for detecting the early stage of serious forms of DVT using the EMG signals with vibration stimuli on a target muscle with the CNN algorithm. The feasibility of this method was tested before and after the induction of DVT (venous stasis) using an acute DVT pig leg model that was similar to completely obstructive symptomatic proximal DVT in a human limb. Furthermore, physiologic changes in the early stage of proximal obstructive DVT were also investigated, including muscle perfusion pressure (PP), intracompartmental pressure (IP), and muscle and soft tissue stiffness in the pig leg.

## Materials and methods

### Animal preparation

The study was approved by the Institutional Animal Care and Use Committee of Korea University College of Medicine prior to the experiment (KOREA-2019-0101-C1). The study was carried out in compliance with the Animal Research: Reporting of In Vivo Experiments (ARRIVE) guidelines. The experiments were also performed in accordance with relevant regulations and guidelines. Four conditioned three-way (Landrace × Duroc × Yorkshire) crossbred pigs (4 males) were selected for our experiment. Following our pilot study about classifying the multi-class time points, the experiments were performed with 8 hind limbs of 4 pigs (Power = 0.8; effect size d = 1.15; α = 0.05). Because both legs were in the same experimental condition and changes within a leg were observed without any additional intervention, the 8 legs were considered as separate cases based on the previous study [36]. Each pig was rested for 3 days in a comfortable cage with easy access to water except, for eight hours before the experiment. The experimental setup was designed based on previous our study [37]. The animal preparation was done similarly reported in our previous study [37]. Briefly, sedation was done using the cervical intramuscular injection of Azaperone (5 mg/kg), Alfaxalone (4 mg/kg), Xylazine (2 mg/kg), and Atropine (0.05 mg/kg). Then, the intravenous catheter was placed on a dorsal ear and NaCl (0.9%) was infused for fluid replacement. Xylazine (0.5 mg/kg) and Alfaxalone (1.5 mg/kg) were injected intravenously for inducing anesthesia. Then, deep anesthesia was maintained by inhaling isoflurane gas. The central blood pressure was continuously monitored using a heparinized 24-gauge catheter (Jelco® IV catheter radiopaque, Smith Medical, UK) inserted in the carotid artery. The catheter was connected to a pressure-transducing device (AMK 150®, Ace Medical Co., Korea Rep.). A heparinized 16-gauge catheter (Jelco® IV catheter radiopaque, Smith Medical, UK) was placed into the intermuscular plane between the peroneus muscles and tibialis anterior muscle under ultrasonographic guidance for monitoring the IP of the anterior compartment of the pig leg.

### DVT surgery and data acquisition

**Fig 1:**
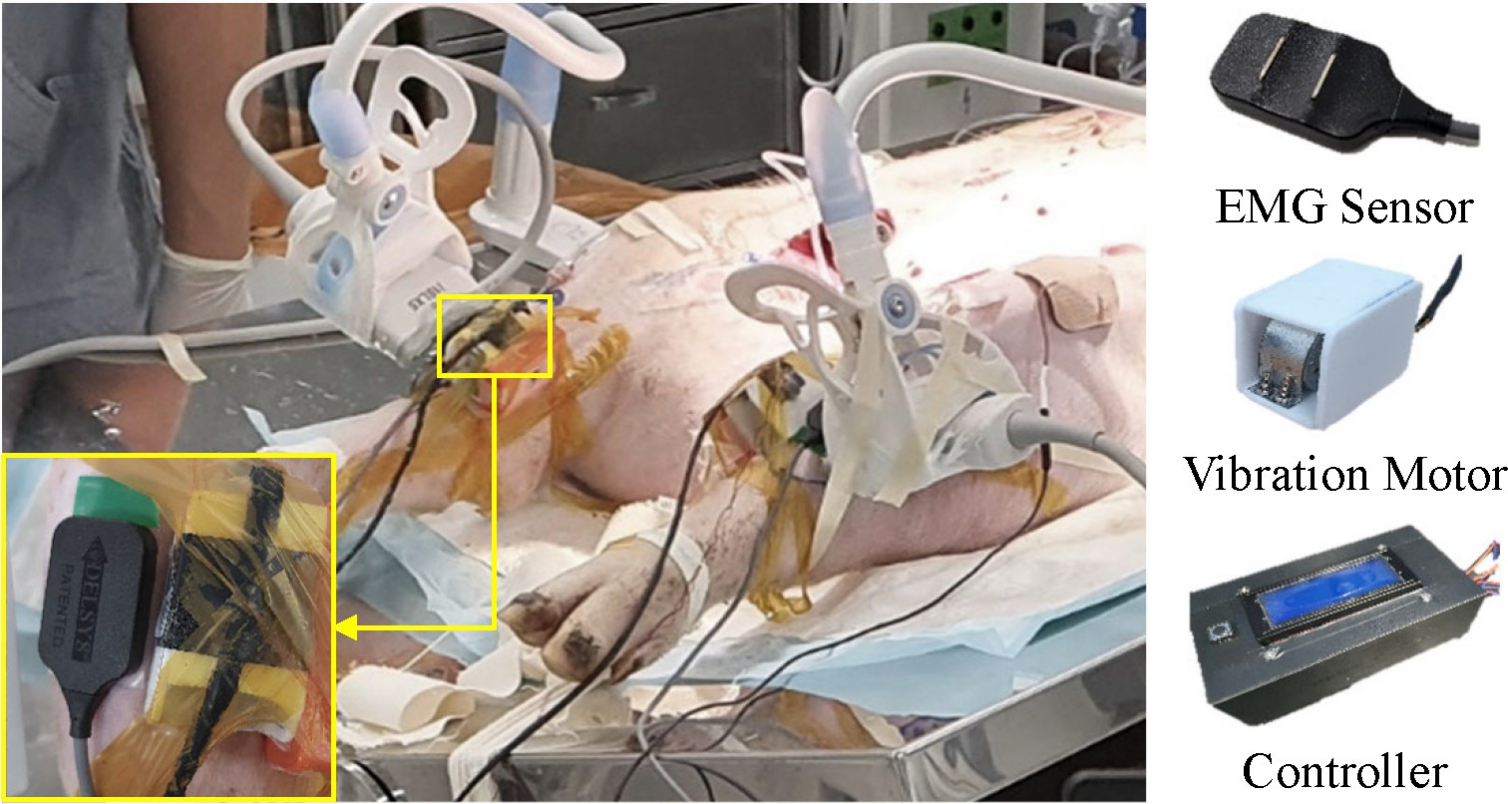
Experimental setup. The EMG sensor and vibration motor were attached to the tibialis anterior of the left and right leg, respectively. Each vibration motor was controlled by the Arduino board.

Each pig was placed, in a supine position, on a surgical table. Both hind limbs were maintained in an extended position by an elastic band (Fig 1). A 12-cm sized skin incision was made along the inguinal line in both hind limbs. After a deep dissection, the femoral vascular bundle was exposed between the adductor muscle and vastus medialis muscle. The femoral vein was carefully dissected after the incision of the inguinal ligament. In the retropelvic space, the common iliac vein (CIV) was identified and tied by a rubber sling to induce venous stasis of the pig’s hind limb. To block superficial veins around the thigh, the proximal thigh was also tied by an elastic band. The elastic band’s tension was adjusted to block superficial venous flow without affecting the femoral arterial and other deep vein flow. During our pilot study for developing our animal model, any changes did not occur in the pigs’ limbs even after 8 hours of common iliac vein ligation; nevertheless, most occlusive proximal DVT induces symptoms such as swelling and cyanosis in human extremities [8,38–42]. That means that it was not enough to induce a similar venous outflow blockade to completely obstructive symptomatic proximal DVT of humans with only common iliac vein ligation because pigs had better-developed collateral venous channels than humans [43]. Additional occlusions of superficial veins were needed to make symptoms in the pig’s leg. Indeed, despite the superficial veins being occluded by an elastic band, the symptoms (swelling and cyanosis) slowly progressed after the blockade of the common iliac vein and superficial veins. That means that other venous channels except for the common iliac vein and superficial veins were maintained. Our animal model did not indicate an extremely severe form of deep venous thrombosis that has obstructions in both deep and superficial venous outflow, so-called phlegmasia cerulean dolens in humans (the most severe form of DVT) because the significant changes in the IP and tissue PP like phlegmasia cerulean dolens were not seen [39,41]. Because these are very similar conditions to completely obstructive symptomatic proximal DVT in humans, our model is suitable for this study.

**Fig 2:**
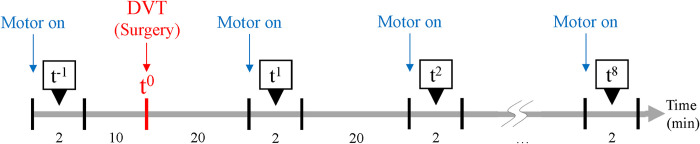
Experimental protocol. The EMG signals were acquired for 2 min before and after the DVT surgery. The experimental period was a total of 188 min.

A commercial ultrasonography machine, Aplio i800 (Canon Medical Systems, Japan) was used to confirm the blockage of the common iliac vein and superficial venous flow of the left and right hind limb using a Doppler mode with the 14L5 linear probe at 11 MHz frequency, and measure the shear modulus of elasticity of the tibialis anterior muscle at each time point (Fig 2). In the present study, we focused on investigating early physiologic changes of proximal obstructive DVTs that are more serious because a prompt diagnosis of them is clinically more critical and urgent before emboli formation. Thus, the blocked blood flow observed in ultrasonography can be a good surrogate for occlusive proximal DVTs. In the shear wave elastography (SWE), the i18LX5 linear probe at 15 MHz frequency was used to acquire SWE images of each hind limb.

The IP, PP, shear elastic modulus (SEM), and EMG of the tibialis anterior muscle were acquired before and after the DVT-inducing surgery using the experimental protocol in Fig 2. The EMG signal was measured every 20 min from just after venous flow blockade up to about 2 and half hours to observe possible EMG signal changes due to aforementioned physiological events associated with venous congestion in extremities related to DVT and investigate the neuroelectric change in the early period of DVT in our animal model. As seen in Fig 2, first, 10 min before the DVT surgery, the EMG signals (t^−1^) were acquired with the vibrating stimulation for 2 min as a baseline measure. Then, 20 min after the DVT surgery the EMG signals were also acquired with the vibrating stimulation for 2 min. This acquisition was repeated 8 times (t^1^–t^8^) to investigate EMG patterns in the time domain.

Prior to collecting the EMG signals in the presence of vibration stimulation, the IP and SEM were also collected without vibration stimuli. As mentioned above, the IP was recorded from the monitor, and PP was quantified by subtracting the IP from the diastole blood pressure. For the SEM, the tibialis anterior muscle of both hind limbs of the pig was selected as the target muscle. The muscles were localized according to ultrasonography. Each ultrasonography probe was placed longitudinally to the belly of the tibialis anterior muscle and angled at 90 degrees to the skin surface. Constant position and contact pressure were maintained throughout the experiment using an adjustable smartphone holder fixed on the experimental table and the 3D-printed probe holder (Fig 1). In the SWE mode, the three random circular regions of interest (ROIs) with a 5-mm diameter were set in the color-coded box presentation (2 cm × 2 cm) immediately after each ultrasonographic image was acquired. After, the SEM at each ROI was calculated automatically in the ultrasonographic machine. The averaged SEM of the three ROIs from at least 5 images was calculated for further analysis.

The vibration stimulation was performed by a weight (MB1103W) and coreless DC motor (MB2607-0335, Nury Electric Co., Ltd.), and it was controlled by a customized controller using an Arduino board (Fig 1). The power supply of the vibration motor used in this study was 3.0 v, which was similar to the power supply needed for the motor used in a cell phone (3.0 v). The vibration frequency was measured before the DVT surgery, and it was 40–50 Hz. The amplitude range of the vibration motor was 0.1–0.6 mm. The vibration stimulator was controlled with the same voltage, but there was a slight difference in frequency due to the attachment pressure and the skin condition of each leg. However, throughout the experiment, each leg had the same pressure from the vibration stimulator by monitoring the Force Sensing Resistor (FSR) value. A vibration stimulator and a bipolar surface electrode (Bagnoli, Delsys, Inc. Natic, MA) were placed on the tibialis anterior muscle of the left and right leg, respectively, for data acquisition (Fig 1). A reference electrode was placed over the Femur. Prior to the electrode placement, the skin was shaved and cleaned with an alcohol swab. When the vibration stimuli were on, the EMG signals were recorded by a customized LabVIEW (National Instruments, Austin, TX) program, with a sampling rate of 1,000 Hz. After each experiment, an intravenous injection of potassium chloride of 2 mmol/kg was performed for euthanasia.

### Preprocessing and feature extraction

The data preprocessing and feature extraction were performed according to the method which showed better performances in previous studies about myoelectric interfaces [35,44,45]. In the method, the acquired EMG data were sectioned into 400-sample segments with 200 overlapping samples. Each segment was then processed independently to calculate the spectrogram and perform normalization. In each pig, normalization was performed on each left and right leg. Each spectrogram was extracted using a 256-point fast Fourier transform using a Hamming window and 184-point overlap. To reduce the negative impact caused by using the Hamming window, overlapping was used. The spectrogram was calculated as 129 (1–1,000 Hz) different frequencies × 3-time bins (1–256, 73–328, and 145–400 samples). The first 95 points in the spectrogram of the frequency were then used [35,45]. Thus, the size of each spectrogram was 95 × 3 (frequency × time bins). Prior to performing a principal component analysis (PCA), the maximum-minimum normalization (in the range of 0 to 1) was applied to the spectrograms [35,45]. The 1st and 99th percentiles in the spectral intensity were considered the minimum and maximum values, respectively [45]. Because the maximum-minimum normalization showed a better performance than the Z-score normalization in the previous study [46], the maximum-minimum normalization was applied in our study.

**Fig 3:**
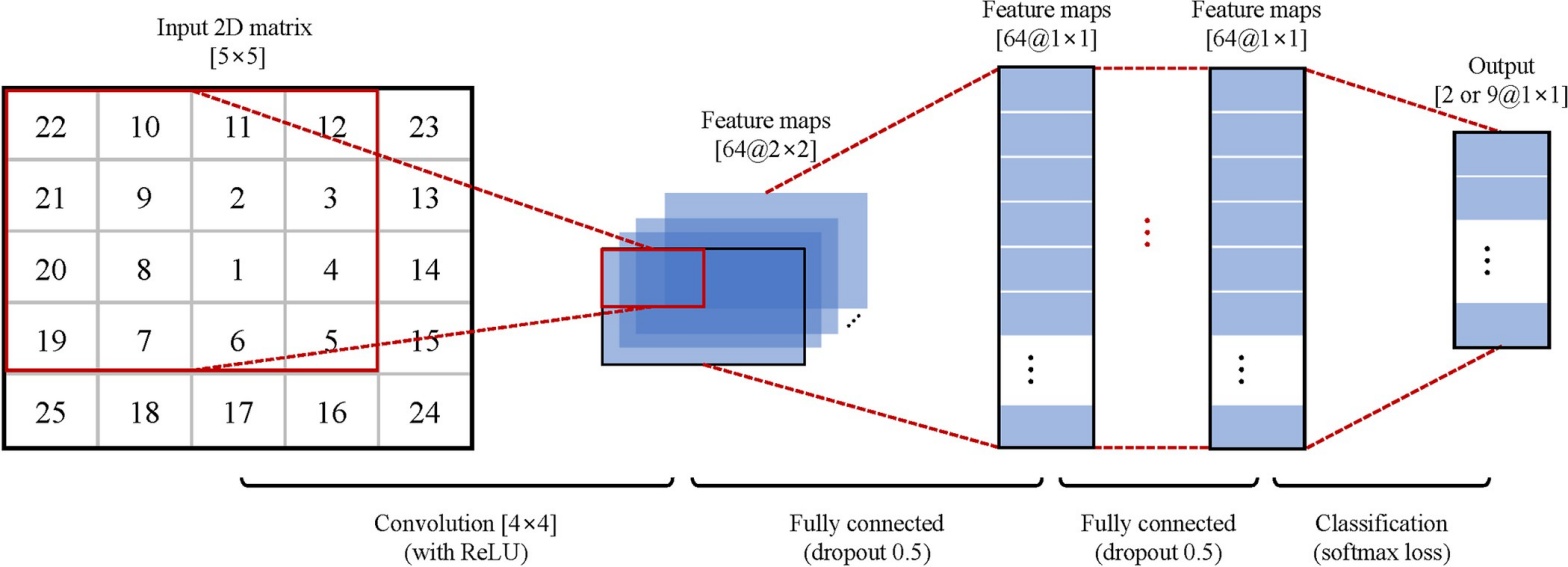
Designed CNN architecture. The 25 PCs were extracted and reshaped into the 5 × 5 matrix with the most significant PC positioned at the center. Then, 64 filters of 4 × 4 and 2 × 2 sizes convolved with the PCs and fully connected for classification.

To apply PCA to improve computational efficiency and performance, the spectrograms were normalized and vectorized to a size of 285. PCA was used to reduce the dimensionality of the spectrogram while retaining useful information [35,45]. The PCA was only calculated on all the segments across all the times in the training set (vectorized and normalized spectrogram matrices × the number of segments). The scores of the first 25 principal components (PCs) were used as input for the CNN classifier. The weightings of the contributions of the principal components to each segment are then used for the classification [45]. Based on the previous studies [45], the first 25 PCs were sufficient to achieve higher classification accuracy. Therefore, 25 PCs were used in our study. Hence, each spectrogram was reduced to a dimension of 25. Before inputting the data into the CNN classifier, the resultant matrices (25 PCs) were rearranged into 5 × 5 matrices. In addition, the PCs were rearranged such that the most significant PC was positioned at the center to optimize the use of CNN (Fig 3). As a result, the major PCs were positioned at the convolving filters, thereby maximizing their contribution [35].

### Design of CNN architecture

Fig 3 shows the CNN architecture used in this study, which was based on previous studies [35,45,47]. The CNN was composed as follows. The first convolutional layer consisted of 64 filters that were 4 × 4 and 2 × 2 in size. The second layer was the rectified linear unit, which is a nonlinear activation function. It was applied to avoid the vanishing gradient problem [48]. The third layer consisted of two fully connected layers (dropout rate of 0.5 [35]) with a size of 64. Because it showed better performance than 32, 128, 192, and 256 maps in a previous study [49], the number of maps was selected as 64. Furthermore, in the dropout method, 50% of the neurons of each layer were randomly chosen as dropout neurons, and these selected neurons were ignored in the weight update procedures and error propagation. The fourth layer was a softmax loss layer to determine the output. This layer evaluated the cost function based on the normalized exponential function. It output a vector that represented the probability distributions of potential outcomes. The softmax loss layer was set to 2 for binary classification or 9 for multi-class classification, respectively. Through several preliminary tests, the CNN parameters were determined as a batch size of 32, momentum of 0.9, and a learning rate of 0.01. To implement the CNN, the MatConvNet (open-source toolbox for MATLAB) was used, and the computations were performed using the NVIDIA GPU and NVIDIA CUDA deep neural network (cuDNN) library.

## Performance evaluation

To investigate the possibility of classifying the EMG data at the time point, we conducted four offline simulations. First of all, the binary classification for only before and after DVT surgery (t^-1^ and t^1^) was implemented based on the 10-fold cross-validation (CV), which has been widely used in EMG studies [50,51]. In the CV, the segmented data for each time point was divided into 10-folds randomly and without any overlap. Then, the 8-folds were used to train the CNN, the 1-fold was used as the validation dataset, and the remained 1-fold was used to calculate the accuracy, as the test dataset. Each fold was used once for testing, and the classification results were averaged to measure the performance. Second, a leave-one-leg-out (LOLO) CV for binary classification (t^-1^ and t^1^) was performed. Because when considering the practical diagnostic tool, it has to detect without any to-be-predicted information for users. In this stimulation, the CNN was trained with datasets from 7 legs and tested by remained 1 leg dataset. Third, in order to confirm the possibility of classifying the DVT stages, the multi-class classification for all time points (t^-1^ –t^8^) was implemented using the 10-fold CV in each leg. Lastly, the binary classification for investigating the possibility of classifying the EMG signals until 1 hour after the DVT surgery (t^1^ –t^4^) and until 2 hours from 1 hour after the surgery (t^5^ –t^8^) was implemented.

### Measuring vibration stimulation transmission depth

As an additional experiment, although the vibration was similar to the cell phone, to make it clearer about the safety of the vibration stimulation applied to humans, we also investigate how deep the vibration can be transmitted from the vibration motor attached to the thigh skin of pigs. The sample size of the safety test was determined as ​four based on the power analysis (power = 0.95; α = 0.05; d = 10.71). Therefore, the acceleration data was acquired at randomized 4 points in the thigh muscles of the two left and two right legs of two pigs to investigate the effect of vibration stimulation on vibration transmission depending on soft tissue depth. The depth was also measured by the ultrasonography machine, Aplio i800 (Canon Medical Systems, Japan).

### Statistical analysis

To investigate the effect of time on the IP, PP, and SEM, Friedman tests were used since the data distribution was not normal. To investigate whether there were correlations between the IP, PP, and SEM, EMG feature data were used as inputs for the CNN analysis. Here, EMG feature data means the standard deviation of the most significant PC of the EMG, as mentioned above (EMG feature std), and were further analyzed to investigate correlations. Correlations among the aforementioned factors were analyzed using Spearman’s rank tests. Furthermore, in the case of the data for the effect of vibration stimulation transmission depth, the Chi-square test was performed first for checking the normality of the acquired data, and the *t*-test was performed.

## Results

### Vibration transmission depending on soft tissue depth

On average, the vibration was detected at 3.06±0.08 cm deeper from the skin and undetected at 3.89±0.08 cm deeper from the skin. The t-test revealed that there was a significant difference in those cases as p < 0.001.

### IP with the DVT surgery

**Fig 4:**
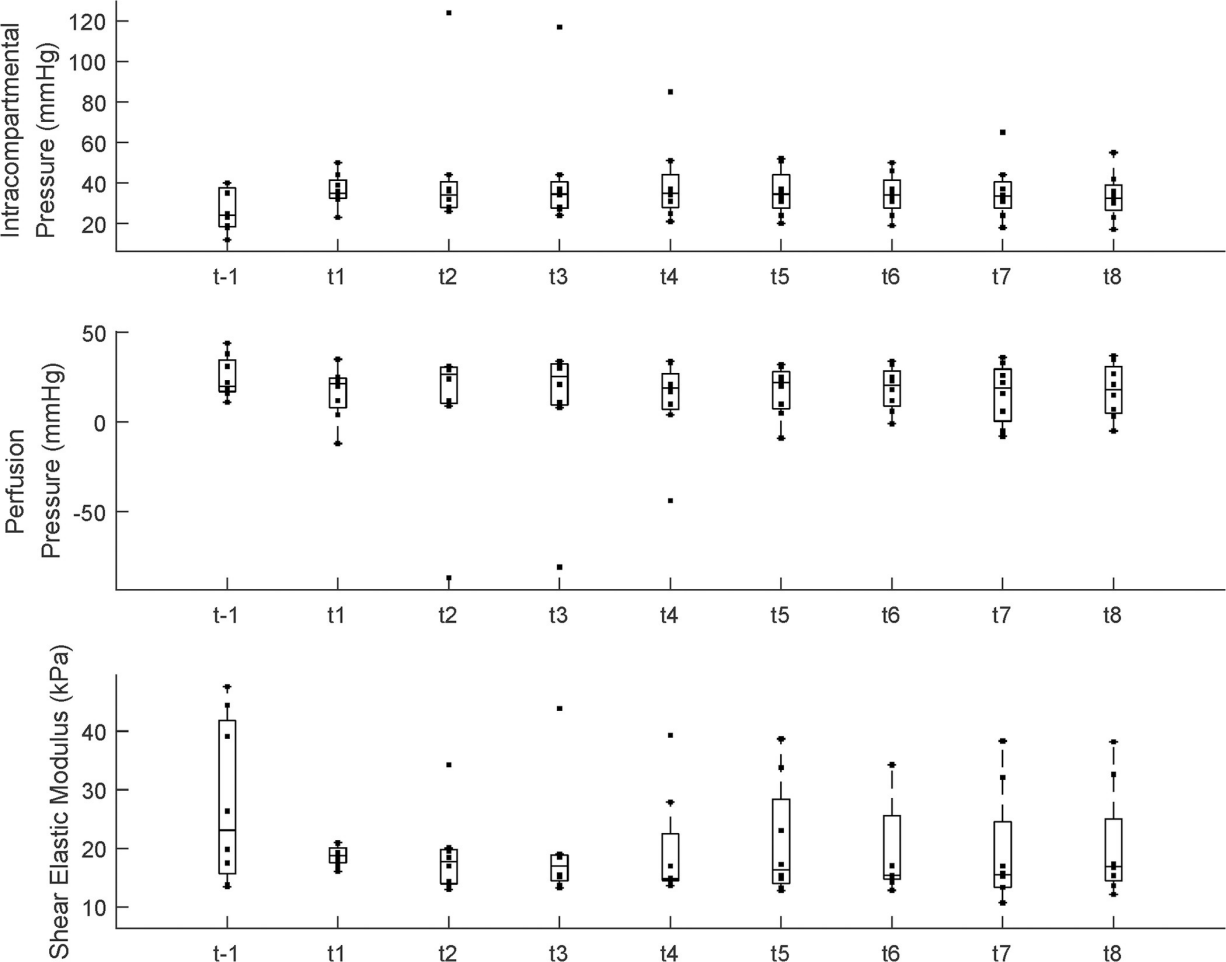
Intracompartment pressure (mmHg), perfusion pressure (mmHg), and shear elastic modulus (kPa). The dot indicates each data point for each group. Each bar plot presents 25^th^ (Q1), 50^th^ (Q2), and 75^th^ (Q3) percentiles of the data. The whisker presents Q3 + 1.5 * (Q3 − Q1), and Q1 − 1.5 * (Q3 − Q1).

Fig 4 presents the IP, PP, and SEM. Time had a significant effect on the IP (p < 0.05); however, post hoc analysis indicated no differences between each time point. Although there is a trend of reduced PP after the DVT surgery, there was no significant difference between each time point. There was no significant effect of time on the SEM; however, there were subject-specific patterns of changes in SEM. In some subjects, the SEM increased, and some maintained a similar value.

### Binary classification for before/after DVT surgery

**Fig 5:**
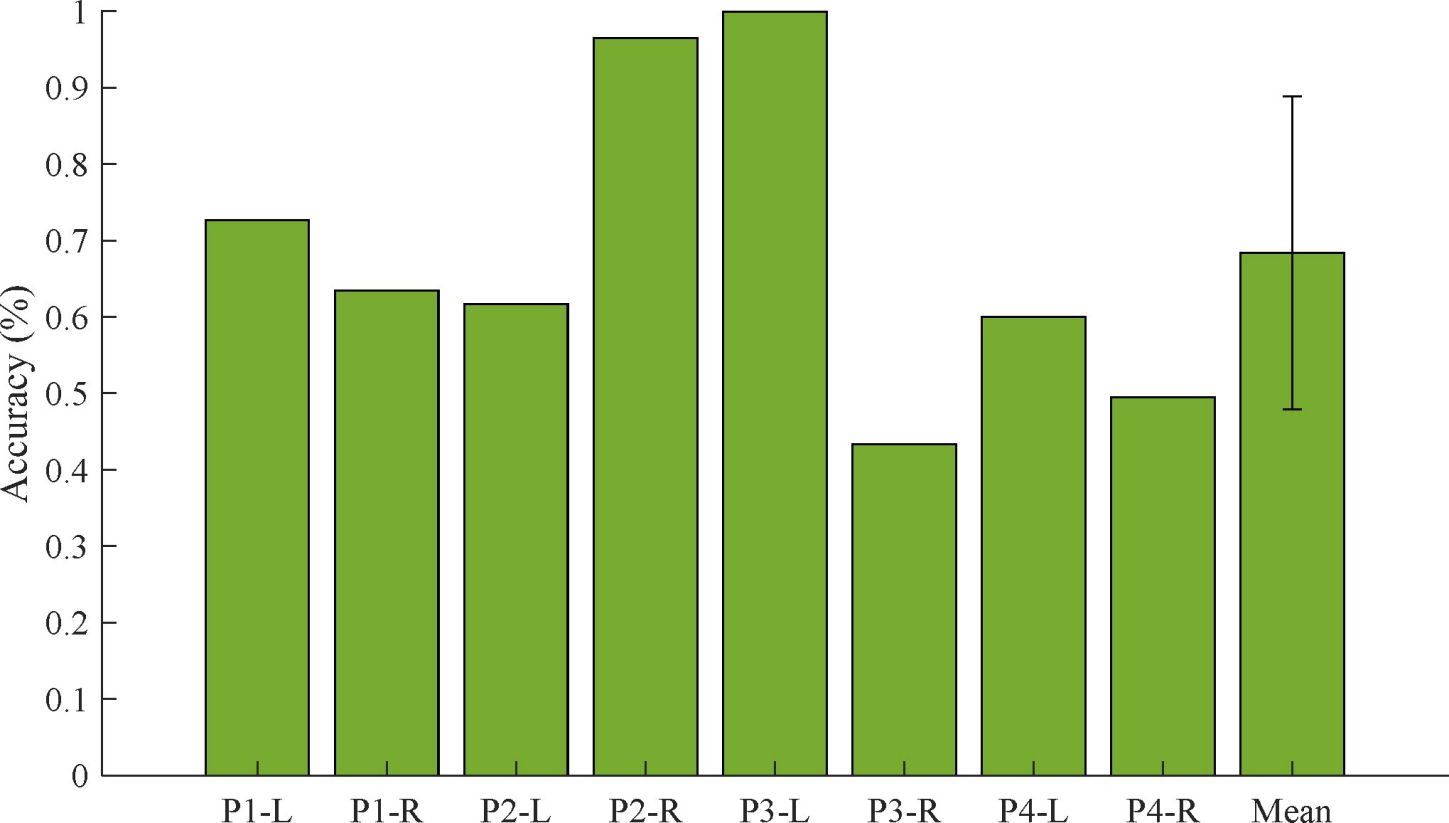
The results of the LOLO-CV for binary classification (t^−1^ –t^1^) in the left (L) and right (R) legs of all four pigs.

In the case of the 10-fold CV for binary classification (t^-1^ and t^1^) that the CNN was trained and tested in only each leg’s EMG data, was calculated as the average accuracy of 100% (in the left and right legs of all pigs). Interestingly, the CNN method classified the test dataset as correct labels, perfectly. However, in the case of the LOLO-CV, the CNN method classified the test dataset within lower accuracies. Fig 5 shows the average results of LOLO-CV for binary classification. The accuracies were calculated as 72.7%, 63.4%, 61.7%, 96.5%, 99.9%, 43.3%, 60.0%, and 49.5%, respectively. The total average accuracy was 68.4±20.5%.

### Multi-class classification of the all-time points

**Fig 6:**
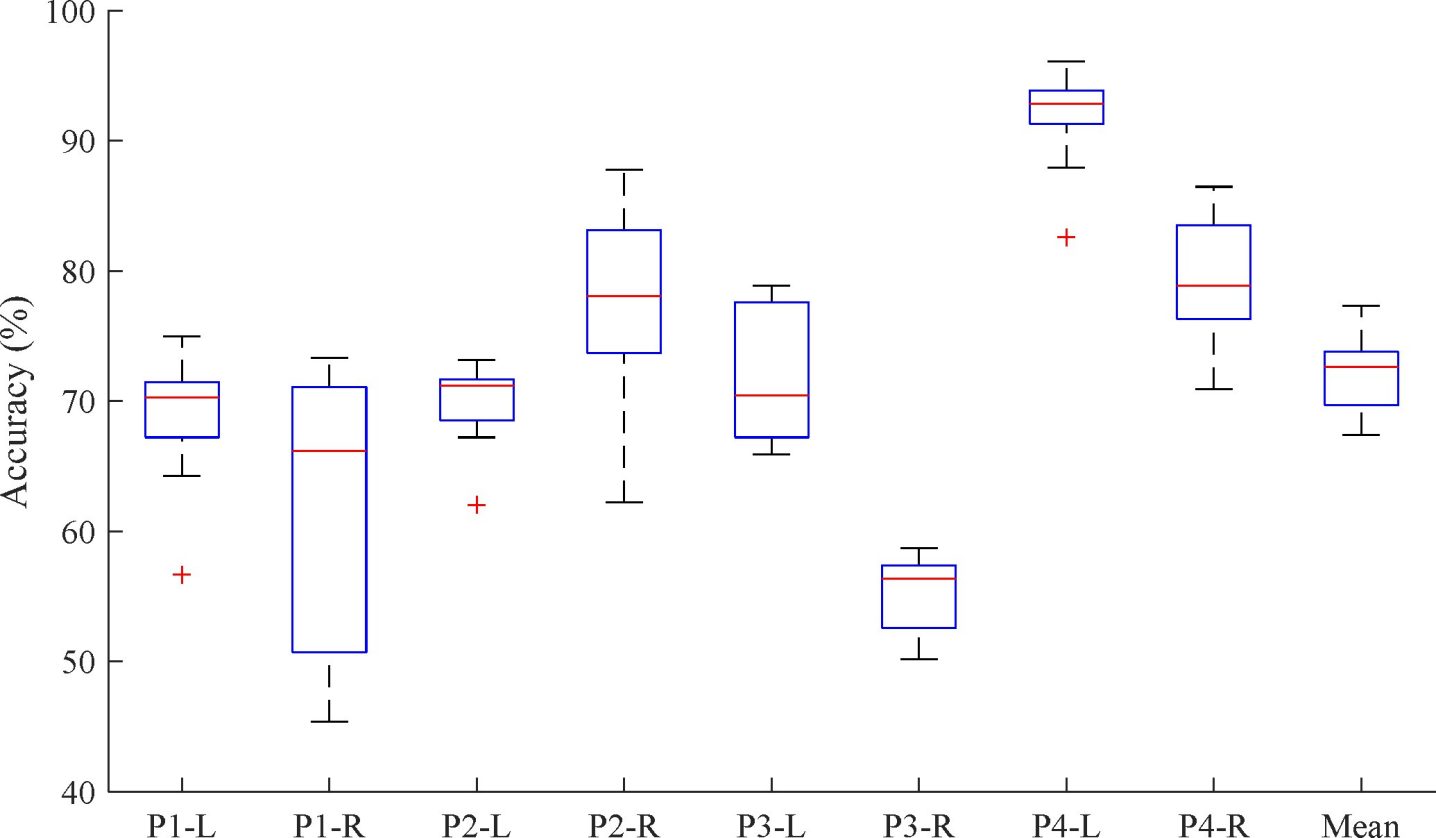
The results of the cross-validation within multiclass classification at all-time points (t^−1^ –t^1^).

**Fig 7:**
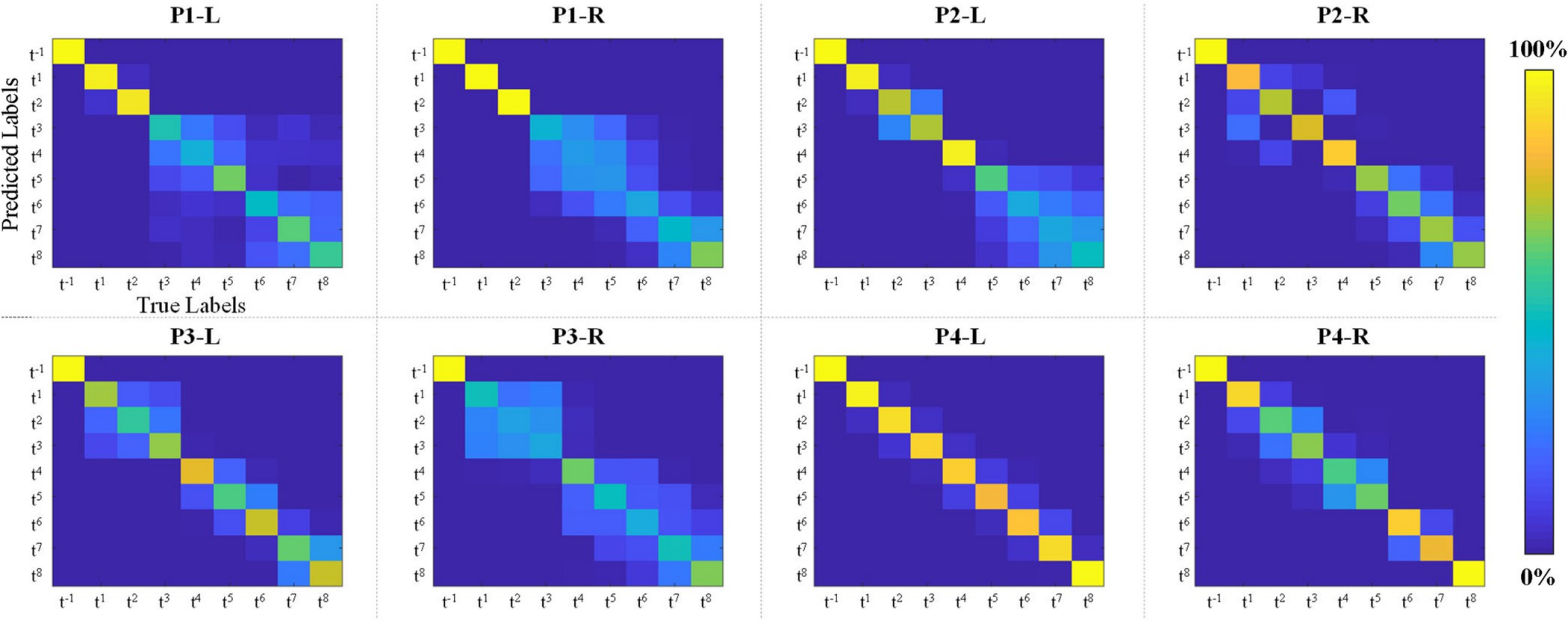
Confusion matrices in each case for multiclass classification. The y-axis indicates the predicted labels and the x-axis indicates the true labels from the CNN classifier in each case.

Fig 6 shows the average results of 10-fold cross-validation with multiclass classification. In Fig 6, each bar represents the average accuracy with a standard deviation for the classification (t^−1^, t^1^, …, and t^8^) for each case. The accuracies were calculated as 68.5±5.1%, 62.5±10.0%, 69.7±3.2%, 77.1±8.0%, 71.9±4.9%, 55.2±3.1%, 91.9±4.0%, and 79.2±5.1%, respectively. The total average accuracy was 72.0±11.9%. To clarify the classification performance across all time points, we also derived the confusion matrix. The confusion matrix is a specific table layout that enables visualization of the classification results. Fig 7 shows the derived confusion matrix by the 10-fold CV for the 9 classes (t^−1^, t^1^, …, and t^8^). In Fig 7, the x-axis indicates the true labels and the y-axis indicates the predicted labels from the CNN classifier in each case. In each confusion matrix, t^−1^ and t^1^ were classified with higher accuracy than t^2^ –t^8^, generally. However, other time points were not classified with high accuracy. In particular, for the right leg of Pig #1, the performances for t^3^ –t^7^ were very low. Furthermore, in the right leg of Pig #3, t^1^ and t^3^ were not classified. Consequently, based on the confusion matrix (Fig 7), the classification accuracies decreased starting from approximately 1 hour after the DVT surgery (t^4^ or t^5^).

**Fig 8:**
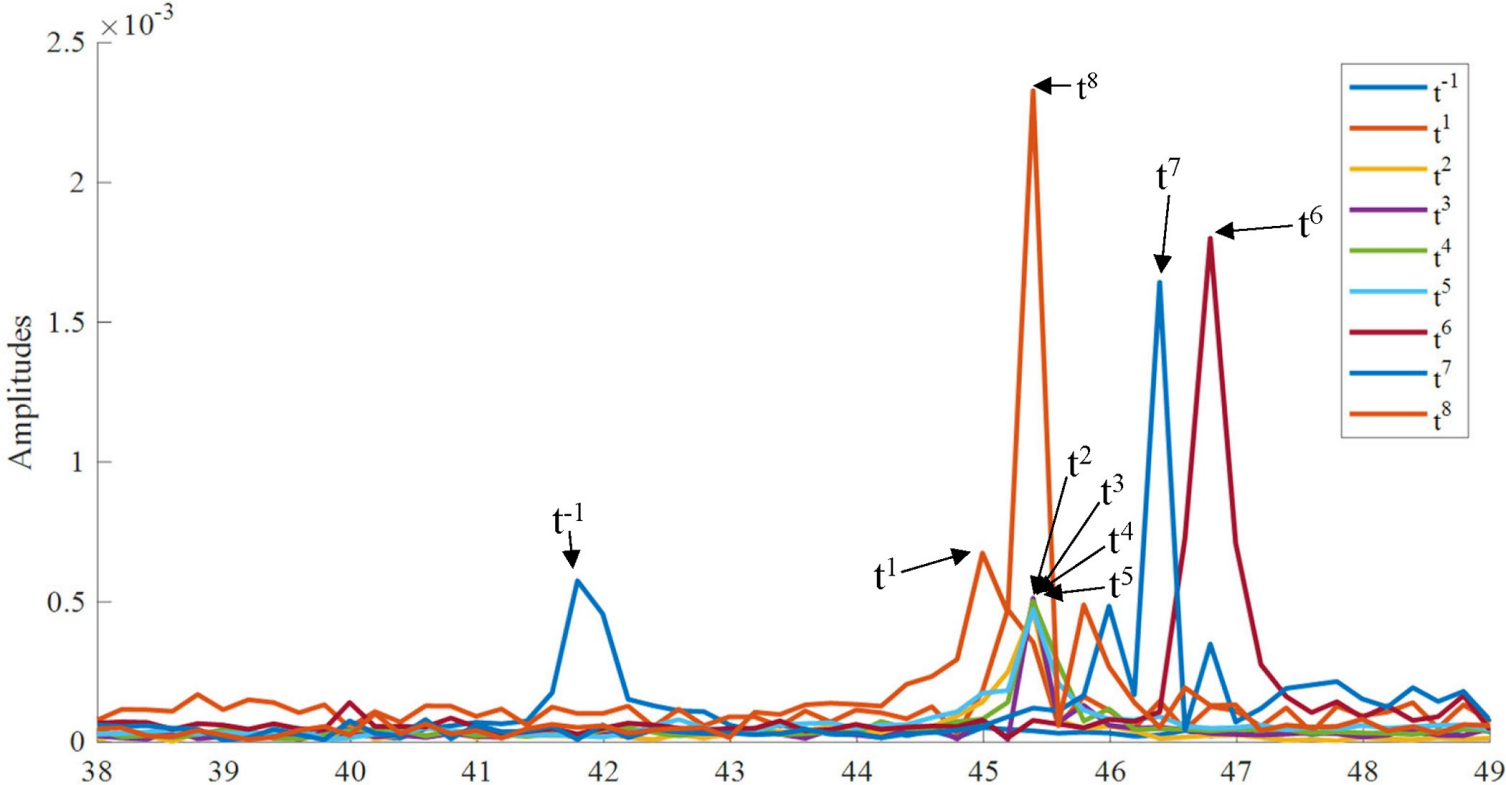
The representative fast Fourier transform results of acquired EMG signals (the right leg for Pig #4). The peak after DVT surgery was higher than before DVT surgery.

To make it clearer about the reason for the results of classification, the fast Fourier transform (FFT) was used for the EMG signals. Fig 8 presents the representative FFT results of the acquired EMG signals for the right leg of Pig #4. In Fig 8, the amplitude before DVT surgery (t^−1^) peaked at a lower frequency than after DVT surgery (t1 –t^8^).

### Hourly binary classification for after the DVT surgery

**Fig 9:**
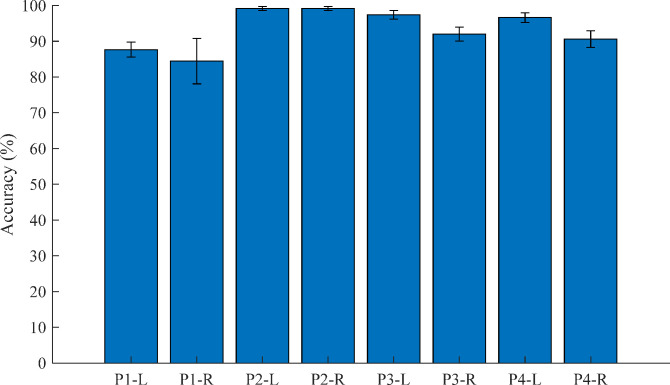
The results of the cross-validation within binary classification for 1 hour and 2 hours after the DVT surgery (t^1^—t^4^ and t^5^—t^8^).

Fig 9 indicates the results of the 10-fold CV after the DVT surgery (t^1^ –t^8^). The accuracies for t^1^ –t^8^ were calculated as 87.7±2.1%, 84.4±6.3%, 99.2±0.6%, 99.2±0.6%, 97.4±1.2%, 92.0±2.0%, 99.6±1.4%, and 90.6±2.3%, respectively. The results indicated that the signals were classified until 1 hour after the DVT surgery (t^1^ –t^4^) and until 2 hours from 1 hour after the surgery (t^5^ –t^8^) with high accuracy.

### Correlation between measures

**Fig 10:**
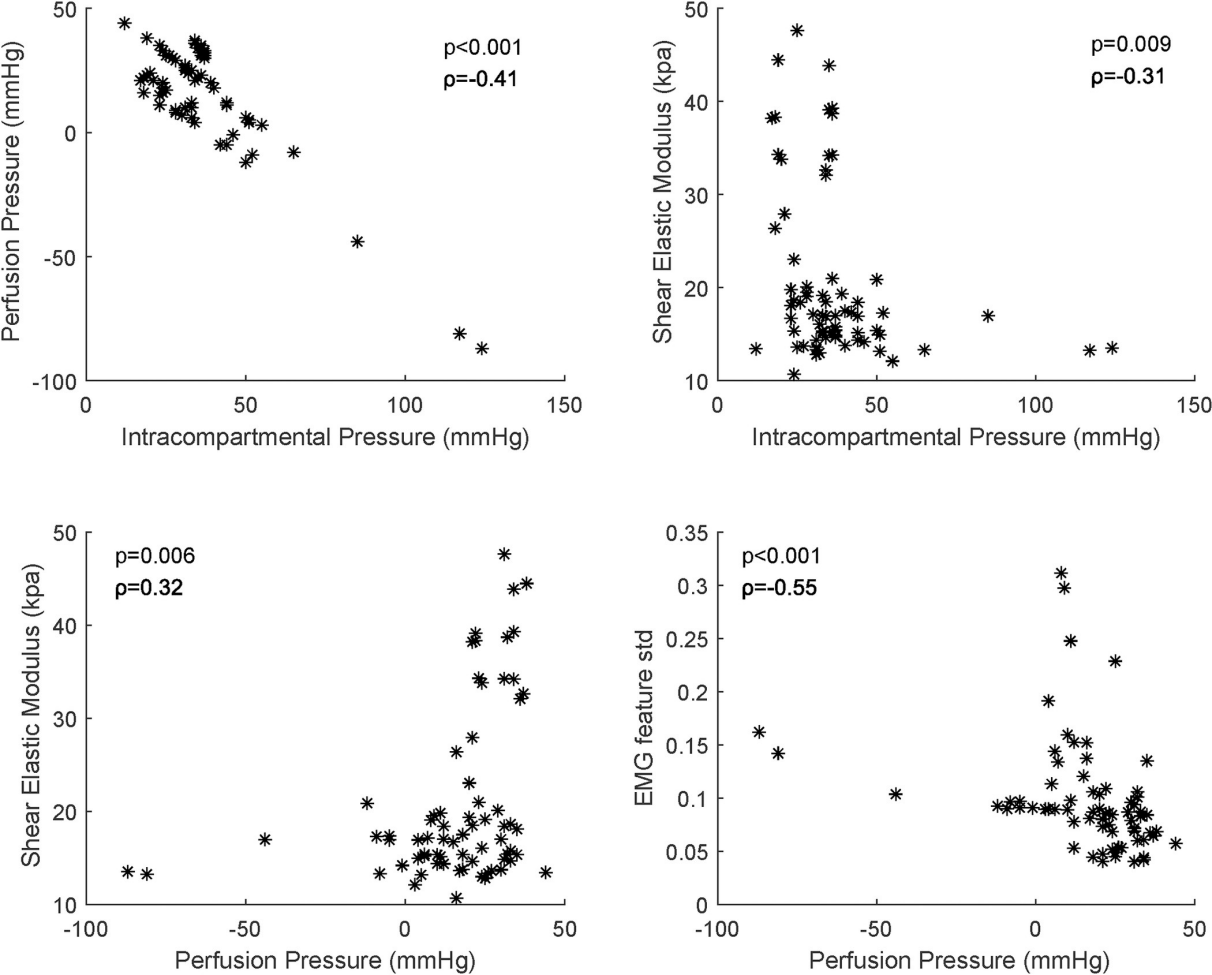
Significant correlation between IP and PP, IP and SEM, PP and SEM, and PP and EMG feature std.

Fig 10 indicates the correlation between IP and PP, IP and SEM, PP and SEM, and PP and EMG feature std. There were significant correlations between PP and IP (p < 0.001, ρ = −0.41, see Fig 10), SEM and IP (p = 0.009, ρ = −0.31), SEM and PP (p = 0.006, ρ = 0.32), and EMG feature std and PP (p < 0.001, ρ = −0.55).

## Discussion

In this study, we developed a novel method to detect physiological differences before and after the induction of proximal obstructive DVT using EMG signal processing in the presence of muscle vibration with CNN-based classification. The results demonstrated that this method can classify DVT with high accuracy, which helps to recognize completely obstructive symptomatic proximal DVT promptly with our EMG monitoring even before symptoms such as swelling and tightness appear. Our findings and approach can be a basis for developing technologies for early DVT detection.

Physiological events associated with venous congestion in extremities related to DVT can change soft tissue stiffness and EMG signals. If the venous flow becomes sluggish or blocked completely, soft tissue would swell and become stiff due to venous congestion. After that, venous congestion would result in increased IP and muscle stiffness. Finally, the increased compartment pressure would cause decreased tissue PP and muscle ischemia, and the ischemic muscle would change the EMG signal successively [52–55]. Because the subject variation and patterns were different among the pigs in this study, we were not able to find any significant values for each metric. However, some pigs showed an increase in SEM with an increase in the IP after the surgical induction of DVT (Fig 10). While it is expected to observe a significantly negative correlation between the PP and IP, the results of the negative correlation between the IP and SEM, and the positive correlation between the PP and the SEM are somewhat ambiguous. In a previous study, a significant positive correlation was found between the shear wave speed and IP in compartmental syndrome (CS) in a dead turkey model [56]. However, this result may be related to the difference in the model and the cause of the pathology. The changes in the muscle stiffness and physiology in our DVT model might have been due to the venous congestion, while the changes in muscle stiffness in CS were due to the direct swelling of the muscle caused by the increase in the IP. Thus, there would be a more distinct positive correlation between the IP and the SEM. Therefore, differently from CS, since the IPs in DVT were indirectly changed by venous congestion, and the changes were various in our cases, the SEM might not be suitable for early detection of DVT.

With our experimental results for binary (before and after DVT) and multi-class (all time points) classification, the CNN-based classification results showed substantially higher accuracy than the chance levels (i.e., 50% for 2-class classification and 11.1% for 9-class classification). Thus, these results might be associated with changes in the EMG signals due to the changes in skin and muscle stiffness and potential neural changes induced by the onset of DVT when the same vibration stimuli were applied. Although the number of subjects was determined by the power analysis, more studies may be needed representing various conditions to generalize our findings. Despite this limitation, this study is useful in demonstrating the potential to detect such changes using the combined vibration stimuli and EMG signals using the EMG signal-based CNN classification.

Furthermore, we applied CNN to classify the stages before and after DVT because it can be used without separating a lot of steps such as feature extraction, feature reduction, and selection [57]. It also showed substantial performance in other fields of research, such as biosignal processing and medical image processing [57,58]. In particular, CNN was recently used to automate the detection of coronary artery disease (CAD) based on electrocardiogram (ECG) signal processing [58]. The experimental result showed that CNN can differentiate between abnormal and normal ECG signals with an average accuracy of 95%. The result demonstrated that CNN can be reliable and efficient in extracting features and characterizing the input ECG signal. From the current study, it is difficult to confirm the feasibility of using CNN for this application due to a lack of information on the EMG-based DVT diagnosis. However, according to the previous study in ECG classification [58], the CNN method has various advantages in biosignal processing for multi-classification. Therefore, our approach of applying CNN to EMG signals may be suitable for classifying the stages of DVT.

In the results of the binary classification within each leg’s CV, the CNN can classify the EMG data before and after DVT surgery, perfectly. However, in the case of the LOLO-CV, the CNN, which was trained by another leg’s EMG data, can classify perfectly only a few cases (P2-R and P3-L). In general, the accuracy was over the chance level (50%) but in some cases (P3-R and P4-R) even lower than the chance level. It can be interpreted as because there are individual differences in symptoms after the DVT surgery. Moreover, the EMG signal is sensitive to uncertain factors such as skin noise, muscular shape, or thickness [59]. For considering the differences, the correlation analysis between the uncertain factors and DVT symptoms is required, and a combination with the PRML method that can reflect the results of the correlation analysis is essential to consider the low accuracy cases such as P3-R or P4-R. In our study, because there were spatial limitations (banding space, sensor attachment, etc.), measuring the individual factor was limited, and the number of data was limited. However, despite the limitation, our study is meaningful in aspects of confirming the possibility to detect DVT without any to-be-predicted information for users. Notably, our experimental results can provide a basis for developing an early diagnostic tool for DVT using only EMG signals with vibration stimuli. Therefore, to make it clearer, additional experiments will be performed for the correlation through a larger population, then our PCA-CNN method will be advanced by combining the PRML methods to consider the individual factors.

Fig 7 shows the confusion matrices from the 10-fold CV results for all time points for each case. The confusion matrix is a specific table layout that enables visualization of the classification results. In the field of PRML, these have been widely used to confirm the outputs from the classifier. In the confusion matrix (Fig 7), the classification accuracies decreased starting from approximately 1 hour after the DVT surgery. These results may have been associated with physiological changes associated with DVT induction. Further research is required with human subjects for further testing our approach and algorithm.

Applying our methods to patients with DVT risk factors such as obesity, immobility, pregnancy, stroke, trauma, cancer, etc [60] can help physicians to detect DVT automatically without fear of neglecting to perform duplex ultrasonography even before DVT symptoms appear. Because our method of early detecting DVT is realized by detecting subtle EMG changes originating from skeletal muscle [61] associated with vibration stimuli, other pathologic conditions that cause muscle swelling like myositis, intramuscular hemorrhage, and muscle injury might not be separated. However, their distinctive clinical signs, symptoms, and trauma or medical history will not cause misdiagnosis of DVT [62]. At the initial stage of DVT, the thrombus can be very loose. In a previous study [63], muscle vibration therapy was safely conducted with stroke survivors who had a higher chance of further cardiovascular diseases compared to healthy individuals. Our vibration amplitude is comparable to the study. Further, our vibration motor had similar power to that of a commercial cellular phone. According to our further experiment, the realized vibration stimulation was transmitted to a depth of only 3~4 cm from the skin. Because our tool will apply to a human calf that is the most susceptible portion for human DVT, the 3~4 cm of transmission is enough depth to reach calf muscles and less deep than the deep veins [64]. Thereby, our approach could not directly cause thrombus shedding. Our technique for early DVT detection is reasonable to apply to calf muscles because they are the most susceptible muscles to DVT [65]. Therefore, our approach would not cause thrombosis shedding and is safe for human application. Further research on the effect of vibration frequency and amplitude on thrombus shedding would ensure the application of our methods to humans.

In our study, the pigs were homogenous in terms of sex as males to control the effects of sex hormones on neuromuscular responses and muscle-to-fat mass ratio and leg circumference (18~19 cm for the knee and 13~14 cm for the ankle). However, considering practical usage, these individual factors can contribute to significant scatter and washout of our method. For instance, the comparative monitoring of both thighs (i.e., side-by-side comparison) could be important to human application. Therefore, in our further research, comparative monitoring, which uses the intraindividual trained CNN considering the individual factors, will be incorporated into our method and additional experiments will be implemented with a large population.

## Conclusion

In this study, a technology for early detecting proximal obstructive DVT was developed by combining vibration stimuli and EMG signals with the CNN algorithm. And the feasibility of the method was tested using a pig model. Although our experiments were implemented with 8 hind limbs, the experimental results showed that the time points before and after DVT surgery can be classified using combined vibration stimuli and EMG signals. Additional studies on a larger population could generalize the current findings in the future. Moreover, additional experiments investigating various types of vibration stimuli on DVT could be an interesting direction to pursue. Following a large human study in the future, our method and tool can be realized for early detection of DVT and other diseases associated with changes in muscle stiffness due to pathological conditions.

## Supporting information

S1 File Representative raw EMG data (Pig #1 –left leg) for our experiment. The first row (T-1, T1, T2, T3, T4, T5, T6, T7, and T8) means the DVT stages.
(XLSX)

S2 File The cross-validation results used to build the graphs (Figs 5, 6 and 9). (XLSX)
